# Ocular lesions in 1,000 consecutive HIV-positive patients in India: a long-term study

**DOI:** 10.1186/1869-5760-3-2

**Published:** 2013-01-03

**Authors:** Sridharan Sudharshan, Sheikh Kaleemunnisha, Akbar Ashraf Banu, Sankaran Shrikrishna, Amala E George, B Rajesh Babu, Bella Devaleenal, Nagalingeshwaran Kumarasamy, Jyotirmay Biswas

**Affiliations:** 1 Medical Research Foundation, 18, College Road, Sankara Nethralaya, Chennai, 600006, India; 2 YRG Care Centre for HIV/AIDS, Chennai, 600113, India

**Keywords:** HIV, AIDS, Immune recovery uveitis, CMV retinitis, Ocular lesions, CMV retinitis, Ocular TB

## Abstract

**Background:**

Ocular lesions in patients on highly active antiretroviral therapy (HAART) have shown changes in disease prevalence and pattern. Although they have been described in the Western population, there are not many such studies in the HAART era from India. This study aims to present the clinical profile, systemic correlation, and visual outcome in HIV-positive patients in relation to HAART in comparison with pre-HAART Indian studies and current Western data. Ocular findings and systemic correlation in 1,000 consecutive patients with HIV seen at a tertiary eye care center were analyzed. This study uses a prospective observational case series design.

**Results:**

Age range of the patients was 1.5 to 75 years. Ocular lesions were seen in 68.5% of the patients (cytomegalovirus (CMV) retinitis was the commonest). The commonest systemic disease was pulmonary TB. Mean interval between HIV diagnosis and onset of ocular lesions was 2.43 years. CD4 counts range from 2 to 1,110 cells/mm^3^. Immune recovery uveitis (IRU) was seen in 17.4%. Interval between HAART initiation and IRU was 4 months to 2.5 years. Recurrence of ocular infection was seen in 2.53% (post-HAART) and > 20% (pre-HAART). Overall visual outcome showed improvement in about 14.3% and was maintained in 71.6% of the patients.

**Conclusions:**

CMV retinitis is the commonest ocular opportunistic infection in India, even in the HAART era. Newer manifestations of known diseases and newer ocular lesions are being seen. In contrast to Western studies, in our patients on HAART, ocular lesions do not always behave as in immunocompetent individuals. Ocular TB needs to be kept in mind in India, as well as other neuro-ophthalmic manifestations related to cryptococci, especially in gravely ill patients. Occurrence and frequency of various ocular opportunistic infections in developing nations such as India have significant variations from those reported in Western literature and need to be managed accordingly.

## Background

Ocular lesions attributable to HIV [1] are seen in upto 2/3rd of the estimated 2.5 million HIV-positive population [2] in India at some point in their lifetime.

Ocular lesions in patients on highly active antiretroviral therapy (HAART) have shown changes in disease prevalence and pattern and have been described in the Western population. Ocular lesions associated with AIDS in India have been described in the past during the pre-HAART era [3-9]. There are not many long-term studies on ocular lesions of HIV-positive patients in relation to HAART, especially in developing nations such as India. We studied the ocular lesions and systemic correlation of 1,000 consecutive patients with HIV in India especially with respect to HAART in comparison with pre-HAART Indian studies and current Western data.

## Results

One thousand HIV-positive patients were included in the study. Their age ranged between 18 months to 75 years (median age, 35 years). Male-to-female ratio was 7:3. The most common presenting complaints were decreased vision and floaters. Overall, ocular lesions attributable to HIV/AIDS were found in 68.5% of the patients (Table 1). Interval between diagnosis of HIV and onset of ocular lesions ranged from 10 days to 10.24 years (mean, 2.43 years). CD4 counts range from 2 to 1,110 cells/mm^3^ (mean 270.82 + 213.36, median 224.50) at the time of inclusion into the study. Mean follow-up was 67 months (range between 1 month and 11 years), and median duration of HIV disease was 360 days. More than 65% of the patients had at least 1 year follow-up. About 51.3% of the patients had an underlying systemic disease. Systemic tuberculosis, the most common opportunistic systemic infection in our study, was seen in 43%, of which pulmonary tuberculosis was seen in 38.01% of patients (Table 2). Other systemic diseases included *Cryptococcus* infection, pneumonia, central nervous system (CNS) toxoplasmosis, and others. Out of 48.70% without any underlying systemic disease, 74.74% were without ocular lesions, while 25.26% had ocular lesions attributable to HIV (Table 3).

**Table 1:** Ocular lesions due to HIV in the pre- and post-HAART era

**Ocular lesions due to HIV**	**Jabs et al.**[10]**(pre-HAART)**	**Biswas et al. **[9] (pre-HAART)	**Present study**
Total number of patients	781	100	1,000
CMV retinitis	37	17	248
HIV retinopathy	50	15	43
Optic atrophy	1	1	19
Ocular tuberculosis	-	-	26
Active toxoplasmosis	1	-	28
Herpes zoster ophthalmicus	3	1	22
Panuveitis	-	-	2
Fibrinous anterior uveitis	-	-	16
Cranial nerve palsies	1	1	5
STBRVO, HCRAO	2	-	3
Endophthalmitis (*Leptospira*)	-	-	7
Color vision defects	-	-	1
Pre-septal abscess	-	-	-
Orbital cellulitis	1	-	-
Molluscum contagiosum	-	1	4
SJ syndrome (nevirapine)	-	-	2
ARN	-	1	11

ARN, acute retinal necrosis; CMV, cytomegalovirus; SJ syndrome, Steven-Johnson syndrome.

**Table 2:** Underlying systemic disease associations in patients with HIV

**Systemic disease**	**Percentage (%)**
Pulmonary tuberculosis	38.01
Extrapulmonary tuberculosis	5
Cryptococcal disease	6.39
Pneumocystis pneumonia	2.49
CNS toxoplasmosis	0.66
CMV esophagitis	0.22
Anemia	0.44

**Table 3:** Ocular lesions in correlation with systemic disease

**Ocular lesions**	**With systemic disease**	**Without systemic disease**
	**(*n*(%))**	**(*n*(%))**
CMVR (*n* = 248)	164 (66.13)	84 (33.87)
HIV retinopathy (*n* = 43)	28 (65.12)	15 (34.88)
Ocular TB (*n* = 26)	17 (65.38)	9 (34.62)
Toxoplasmosis (*n* = 28)	17 (60.71)	11 (39.29)
HZO (*n* = 22)	18 (81.82)	4 (18.18)
Others (*n* = 318)	142 (44.85)	176 (55.3)

CMVR, cytomegalovirus retinitis; HZO, herpes zoster ophthalmicus.

**Figure 1:**
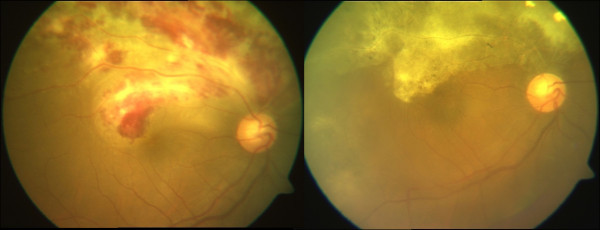
**Composite color fundus photographs showing a case of active and healed CMV retinitis.** Pre- (left) and post-treatment (right) with ganciclovir.

Among patients with ocular lesions, CMV retinitis (Figure 1) was the commonest and was seen in 248 patients (36.2%). All patients with active CMV retinitis were treated with induction therapy with intravenous ganciclovir (5 mg/kg of body weight every 12 h) along with an intravitreal injection of ganciclovir, in most cases followed by maintenance therapy. In our study, two patients with CMV retinitis on HAART presented with neovascularization and vitreous and subretinal hemorrhage and were managed accordingly.

**Figure 2:**
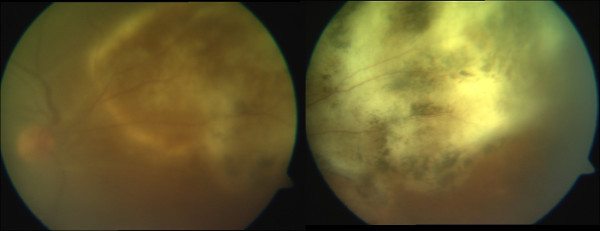
Composite color fundus photographs showing a case of active (left) and healed (right) toxoplasmic retinochoroiditis.

Ocular toxoplasmosis (Figure 2) was the next most common ocular opportunistic infection and was seen in 28 patients (4.09%). Most of them were diagnosed based on clinical features and supportive anterior chamber tap findings, positive polymerase chain reaction (PCR) or antibodies for toxoplasmosis. All patients were treated with combined anti-toxoplasma therapy based on clinical and serological evidence.

**Figure 3:**
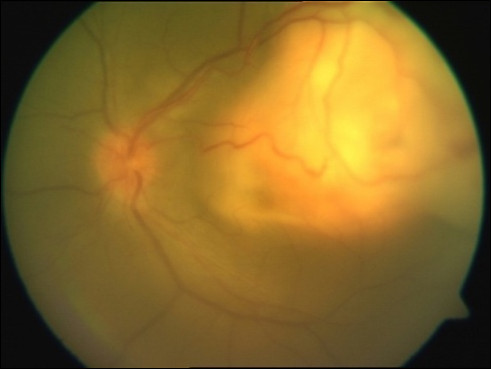
Color fundus photograph showing a case of active choroidal granuloma due to tuberculosis.

Ocular tuberculosis (Figure 3) was seen in 26 patients (3.8% patients), presenting as choroidal tubercles, subretinal abscess, conjunctival tuberculosis, and panophthalmitis. All cases had evidence of pulmonary tuberculosis. Coexistent central nervous system and abdominal tuberculosis were the associations. CD4+ cell counts in patients with ocular tuberculosis were between 14 to 560 cells/μl (mean, 160.85 cells/μl). HIV retinopathy was seen in 6.28% of the patients. Other lesions noted significantly were acute retinal necrosis in 1.6%, progressive outer retinal necrosis in (PORN) in 1.17%, syphilis (1.2%), endophthalmitis (1.02%), fungal corneal ulcer (0.3%), retinal vessel occlusions (0.44%), and anemic retinopathy (0.73%).

**Figure 4:**
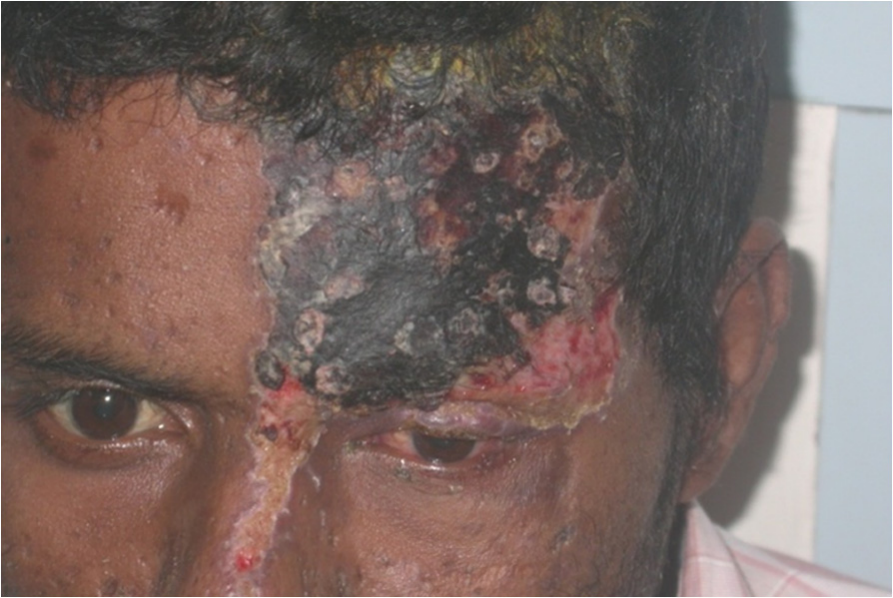
External photograph showing herpes zoster ophthalmicus.

One hundred three patients (15.04%) had anterior segment, adnexal, and/or ocular surface lesions. CD4 count range in this subgroup of patients was 19 to 964 cells/mm^3^. HZO was the commonest, seen in 21.4% of patients with anterior segment manifestations (Figure 4). Others included anterior uveitis in 16 patients (15.57%), ulcerative blepharitis, keratitis, conjunctival microvasculopathy, keratoconjunctivitis, conjunctival squamous cell carcinoma, molluscum contagiosum, episcleritis, Stevens-Johnson syndrome, lid abscess, and herpetic sclerouveitis. Most viral anterior segment lesions had higher CD4 counts of > 250 cells/mm^3^. Significantly, 12 patients (13.3%) were not known to be HIV-positive and were diagnosed based on clinical suspicion.

Neuro-ophthalmic lesions were seen in 61 patients. Optic atrophy, seen in 49.45%, and disk edema, seen in 21.97%, were the most prominent manifestations. Others included optic neuritis in 14.28%, cranial nerve palsies in 9.89%, retrobulbar neuritis in 2.19%, and optic nerve head infiltration and cortical blindness in one patient each. *Cryptococcus* infection was the commonest underlying infection in 66% patients.

Multiple ocular opportunistic infections in the same eye/patient were seen in ten patients. The commonest combination was presence of toxoplasmic and cytomegalovirus retinitis, seen in four patients. Others included ocular TB and CMVR, and herpetic retinitis and CMVR in two patients each, and ocular toxoplasmosis with ocular syphilis and ocular TB in one patient each.

Confirmation of the etiological agent was done in select cases by intraocular fluid evaluation in 8.6% patients with a 72% yield. Specific ocular medical and surgical therapy was administered based on the diagnosis.

The commonest combination regimen of HAART was lamivudine, nevirapine, and stavudine in 84% of patients. Drug combinations were individualized based on response to treatment or development of resistance under care of an AIDS care physician.

When comparing patients on antiretroviral therapy (ART) and those not on ART with respect to the diagnosis of ocular lesions, it was found that 52.2% of patients were on ART at the time of ocular lesion diagnosis, 62% of them had CD4 < 200 while the rest had CD4 counts more than 200. While among patients on ART and without ocular lesions, 31% had CD4 counts less than 200, and significantly, in spite of this, no ocular opportunistic infection was noted even during the period of follow-up.

There were 47.7% of patients who were not on HAART at the time of ocular lesion diagnosis; 69% of them had CD4 counts less than 200. While among patients not on ART and without ocular lesions, 73% of patients understandably had CD4 counts more than 200, while significantly 27% of them had CD4 counts less than 200 (Table 4).

**Table 4:** Comparison of presence of ocular lesions in patients with/without HAART in relation to CD4 counts

	**With ART**	**Without ART**
Ocular lesions (%)	52.2	47.8
CD4 < 200 + OI (%)	62	69
No ocular lesions		
CD4 < 200 (%)	31	27
CD4 > 200 (%)	69	73

Immune recovery uveitis (IRU) was seen in 17.4% of the patients on HAART. Interval between the start of HAART and onset of IRU was 4 months to 2.5 years, and 52% of the patients developed IRU when the increase in CD4 counts was between 100 and 150 cells. Seventeen patients were treated with topical steroids and cycloplegics, one patient was treated with topical and periocular steroid injection, and five patients were treated with an additional short course of oral steroids along with topical steroids.

In our series of patients, immune recovery uveitis was seen predominantly post-CMVR in 88% of patients, while IRU post-tuberculous and post-cryptococcal infection was noted in 6% and 2% of patients with IRU, respectively. Whereas in 4% of patients, there was inflammation post-immune recovery; the cause remained undetermined. Recurrence of ocular infection was seen in 2.53% (post-HAART) and > 20% (pre-HAART) of patients.

Based on the severity of visual impairment, the patients were divided into groups with visual acuity better than 6/12, between 6/12 and 6/60, and worse than 6/60. Pre- and post-treatment (i.e., at presentation and at final follow-up) were analyzed (Table 5). At presentation, 57% had visual acuity better than 6/12, while at final follow-up 58.3% fell in this group; 13.9% had moderate visual impairment, and at the end of treatment, 13% were in this group. At presentation, 29.1% had vision poorer than 6/60, and 28.8% belonged to this group at the final analysis. The visual outcome among various groups, pre- and post-treatment, is given in Table 6.

**Table 5:** Pre- and post-treatment visual acuity: comparison between various groups based on severity of visual impairment

**Visual acuity**	**Pre-treatment (%)**	**Post-treatment (%)**
< 6/12	57.0	58.3
6/12 to 6/60	13.9	13.0
> 6/60	29.1	28.8

**Table 6:** Visual outcome pre- and post-treatment in the various groups

**VA outcome**	**Pre-treatment**			**Post-treatment**		
	**< 6/12**	**6/12 to 6/60**	**> 6/60**	**< 6/12**	**6/12 to 6/60**	**> 6/60**
Improved (%)	12.80	26.30	60.90	80.50	12.80	6.80
Maintained (%)	63.40	10.50	26.10	63.30	10.50	26.20
Worsened (%)	68.90	18.90	12.10	10.60	25.80	63.60

Considering a two-line improvement in Snellen’s visual acuity, overall visual outcome showed improvement in about 14.3% and was maintained in 71.6% of patients. While in 14.1% of patients, vision deteriorated due to complications such as retinal detachment, optic atrophy, macular scarring, and others.

## Discussion

India has a large HIV-positive population when compared to many other countries. There have not been many long-term large-scale studies based on HIV-positive population and ocular lesions in India. In our published series of first 100 patients, 77% of the HIV-positive patients were men in the age group of 20 to 40 years; with national figures ranging from 45.6% to 74.2% occurring due to heterosexual transmission [9], homosexual transmission was only between 5% and 10% in India. Our study also revealed that ocular involvement was more common in children who has acquired the disease due to perinatal transmission (66.7%) and in homosexual patients (60%). Ocular involvement was comparatively less common in patients who had contracted this disease through blood transfusion (33%) or exposure to a commercial sex worker (24.3%).

India also has a unique population of undertreated HIV disease, and they seem to have much lower CD4 counts. Surprisingly, the opportunistic infections seem to have many similarities to the West, with very few differences. Whether long-term exposure to infectious pathogens, or herd immunity, contributes to the tolerance of Indian eyes to opportunistic infections is debatable.

The commonest ocular opportunistic infection even in our study was CMV retinitis as reported in Western literature [9,10]. The next most common lesion was HIV retinopathy (6.28%), which is much lesser when compared to studies in Western countries (50%) [8]. In India, patients infected with HIV do not undergo routine ophthalmic evaluation. They are often referred for ophthalmic examination only if they present with visual complaints. This fact may result in the underestimation of asymptomatic HIV retinopathy or early peripheral CMV retinitis. CMV retinitis was seen even in patients on HAART. Patients on regular HAART and with improved CD4 counts, when treated with systemic and/or intravitreal ganciclovir therapy, showed complete resolution of retinitis without the need for maintenance therapy. More importantly, the number of recurrences was found to be much lesser in such patients, and systemic ganciclovir therapy could be discontinued after resolution of lesions. The progression of retinitis in patients on HAART, even with low CD4 counts, was lesser in Western studies even though they were expected to behave like pre-HAART-era patients. Although the prognosis was better in most patients on HAART and ganciclovir, CMVR was also seen to be presenting with neovascularization, vitreous hemorrhage, and retinal detachment with proliferative vitreoretinal changes, as is seen in an immunocompetent state.

In contrast to the study by Jabs et al. [11], in our study, we found that the progression of retinitis leading to complications was significantly more in patients with lower CD4 counts in spite of regular HAART. In an earlier publication from the same center, low CD4 counts were commonly associated with drug resistance. Forty six percent of the patients experienced clinical failure and developed a new WHO-defined opportunistic infection [12]. We hypothesize that this could be an important contributing factor for low CD4 counts in spite of HAART even in our study.

Involvement of the second eye and the occurrence of retinal detachment in patients who were on regular HAART have been studied by Jabs et al. [13]. Significantly, 88% of the patients with unilateral disease who were on regular HAART did not have involvement of the other eye throughout the duration of the follow-up.

Importantly, a CD4 count limit of 200 had a better clinical correlation with HAART and the occurrence of ocular lesions, with lesions seen in a larger number of patients not on HAART and with low CD4 counts. Interestingly, 27% of the patients were not on HAART and had persistently low CD4 counts for more than 6 months and did not develop any ocular lesion attributable to HIV.

In the Indian scenario [14], although systemic TB is the commonest underlying systemic infection, ocular TB is relatively rare. Ocular TB is found in all CD4 count ranges, and asymptomatic choroidal tubercles are among the commonest manifestations of ocular TB in AIDS. Resolution of systemic and ocular TB with antituberculosis treatment (ATT) is not always concurrent. PCR and HPE methods have been helpful in diagnosis. Clinical profile with histopathological and microbiological correlation of ocular tuberculosis in patients with AIDS has been reported.

Herpetic viral retinitis in our patients on HAART presented with significant inflammatory reactions, more in the form of acute retinal necrosis (ARN) than the classical description of PORN in immunosuppressed individuals. There were 11 patients with ARN in our study, much higher than those in the pre-HAART era.

Shalaby et al. [15] and Kuo et al. [16] have described various presentations of syphilis in HIV-positive patients. Ocular syphilis was found in 1.2% of patients in our study. Two of our patients were serologically positive for syphilis and then were diagnosed to be HIV-positive; suspicion was based on suggestive eye findings and positive syphilis serology [17]. Both of these patients were treated with systemic penicillin along with HAART.

Retinal vessel occlusions have been described in patients with HIV infection [10,17,18]. In our study, we had two patients (two eyes) with superior temporal branch retinal vein occlusion and one patient with CMV retinitis and hemi-central retinal vessel occlusion, all of whom responded well to anti-CMV treatment.

Steven-Johnson syndrome in HIV-positive patients treated with nevirapine has been reported [19] and was seen in our series of patients too. Cryptococcal ocular involvement secondary to intracranial involvement manifesting as papilledema [20,21], optic atrophy, and ophthalmoplegias was noted. Though CMVR was the commonest ocular opportunistic infection even in patients on HAART, cryptococcal infection and related complications were more common in gravely ill patients.

In 72% of patients on whom aqueous tap was done, a confirmed clinical diagnosis could be made based on aqueous fluid analysis by PCR and/or antibody titers. The usefulness of AC tap for PCR and antibody production has been studied by several authors [22,23]. Though AIDS-related lymphomas have been reported to have a better prognosis in the HAART era [24], one patient with non-Hodgkin’s lymphoma in our study developed hard palate necrosis and orbital cellulitis with complete loss of vision.

IRU [25-29] has been extensively studied, and we have seen it in 17.4% of patients on HAART. Nguyen et al. [27] have studied the incidence of immune recovery uveitis in 33 patients with CMV retinitis, and they found six patients with immune recovery uveitis. The incidence rate of immune recovery uveitis was 0.109/person-year in their study. Kempen et al. [28] concluded that patients with IRU have a higher risk of additional morbidity over and above that seen in patients with CMV retinitis. Similar findings were noted in our series of patients. Immune recovery state had led to differences in the way the ocular lesions presented along with drug-induced reactions.

## Conclusions

CMV retinitis is still the leading cause of ocular opportunistic infection in India, and ocular toxoplasmosis is the second commonest. Although HAART reduced the risk of OIs, newer manifestations of known diseases such as in CMV and neovascularization or retinal detachment and newer ocular lesions such as drug-related reactions are being seen in patients on HAART. Ocular opportunistic infections may present with significant inflammatory reaction in patients on HAART. Ocular TB is common in India although not proportional to systemic TB in the Indian HIV-positive population. Paradoxical worsening post-TB infection is also noted in patients on HAART and ATT.

Even in comparison with the previous study in India [9], other opportunistic infections such as ocular toxoplasmosis, ocular tuberculosis, and HZO were found in large numbers in the present study. It could perhaps be due to the increased awareness and referral by physicians in India. This also indicates the need for regular ophthalmic evaluation of all patients with HIV/AIDS irrespective of CD4 counts as ocular lesions are noted in spite of HAART.

Treatment options need to be modified based on local needs and resource constraint settings such as the use of intravitreal ganciclovir. In contrast to Western studies, in our patients on HAART, ocular lesions do not always behaved as in immunocompetent individuals. Ocular TB needs to be kept in mind in India, as well as other neuro-ophthalmic manifestations related to cryptococci, especially in gravely ill patients. Interestingly in India, even with incomplete or irregular HAART, the occurrence of opportunistic infections did not vary much when compared to many other Western studies.

Early recognition and treatment of vision-threatening ocular opportunistic infection and IRU can lead to maintenance/improvement of visual acuity in most patients. Importantly, our study included patients spanning the pre- and post-HAART era with a regular ocular and systemic follow-up using a standard protocol at both the tertiary care eye center and the AIDS care center. Although there are a few limitations as in any long-duration study due to changes in the disease pattern, which have also been studied, our study has shown that the occurrence and frequency of various ocular opportunistic infections in developing nations such as India has significant variations from those reported in Western literature and need to be managed accordingly.

## Methods

The first 1,000 consecutive patients found to be HIV-positive who were seen in the outpatient department of a tertiary eye care institute in India between December 1993 and June 2010 were analyzed. All patients diagnosed with HIV infection who were either referred to the institute for evaluation of their visual complaints or with low CD4 counts/advanced systemic disease were included. Patients with a minimal follow-up of 3 months and/or with at least two follow-up visits to both the ophthalmic clinic and the AIDS care center were the inclusion criteria. Other patients presenting to our clinic who were not originally known to be HIV-positive and were subsequently tested for and diagnosed to be HIV-positive because of suspicious ocular lesions were also included. Complete ophthalmic evaluation findings including thorough history, best corrected visual acuity, external eye examination, clinical features, and fundus evaluation by indirect ophthalmoscopy were analyzed. Fundus fluorescein angiography, ultrasound examination, ultrasound biomicroscopy, and other ancillary investigations such as electroretinogram and optical coherence tomography were obtained in all cases wherever necessary. All these patients had a thorough systemic evaluation at an AIDS care and research center in the city. All patients in our series were screened for associated opportunistic systemic infections such as tuberculosis, syphilis, toxoplasmosis, cryptococcal infection, etc. along with routine blood investigations. Information was recorded in a precoded proforma. CD4+ and CD8+ counts were obtained in all cases at the time of inclusion into the study. The antiretroviral drugs used for the treatment of AIDS and their effect on the disease and on the eye were also analyzed. Lab investigations related to ocular and systemic findings were also analyzed in detail.

### Study design

A prospective observational case series design was used in this study.

## Consent

Institutional review board and ethics committee approval and patients’ consent was obtained.

## Competing interests

None of the authors have any competing interests with the study.

## Authors’ contributions

NK, JB – overview of the study and clinical management of patients. SS – overview, concept, planning, execution, analysis, manuscript and clinical management of patients and coordination. KS, SK, AB – Data collection and entry and analysis. BD, AEG – clinical management of patients and inputs. RB, – clinical management, execution.

## Authors’ information

None of the authors have any financial interests in the study.
